# MFAP2 promotes the progress of esophageal squamous cell carcinoma by enhancing PTGS2 signaling

**DOI:** 10.7150/jca.106659

**Published:** 2025-06-18

**Authors:** Zidong Chu, Pengyu Pan, Shaoyan Qi, Sujuan Fang, Zhe Zhang, Zhenguo Cheng, Baisui Feng

**Affiliations:** 1 Department of Gastroenterology, The Second Affiliated Hospital of Zhengzhou University, Zhengzhou, China.; 2 Department of Intensive Care Unit, The Second Affiliated Hospital of Zhengzhou University, Zhengzhou, China.; 3 National Centre for International Research in Cell and Gene Therapy, Sino-British Research Centre for Molecular Oncology, State Key Laboratory of Esophageal Cancer Prevention & Treatment, School of Basic Medical Sciences, Academy of Medical Sciences, Zhengzhou University, Zhengzhou, China.

**Keywords:** MFAP2, esophageal squamous cell carcinoma, esophageal adenocarcinoma, PTGS2, macrophage

## Abstract

Esophageal squamous cell carcinoma (ESCC) is one of the most prevalent malignancies, accounting for over 85% of all esophageal cancers worldwide and over 90% in China. Due to the absence of effective early diagnostic tools and therapeutic approaches, the 5-year survival rate remains below 30%. Thus, it is crucial to further investigate the molecular mechanisms underlying ESCC. By analyzing differentially expressed genes in esophageal adenocarcinoma (EAC) and ESCC, we identified 127 genes that were significantly upregulated in ESCC and enriched in extracellular matrix organization. Notably, MFAP2 (microfibril associated protein 2), a matrix-related molecule with unclear function, was found to be highly expressed in ESCC in both the TCGA database and our RNA sequencing data. Its elevated levels were associated with cancer progression. Western blot, immunofluorescence, and immunohistochemistry revealed that MFAP2 protein was highly expressed in ESCC and predominantly distributed in the extracellular matrix, cytoplasm, and partially in the nucleus. *In vitro* functional experiments demonstrated that overexpressing MFAP2 had no significant effect on cell proliferation but inhibited cell migration and invasion. *In vivo* xenograft assays showed that MFAP2 enhanced the growth of the KYSE-450 cancer cell line, though no statistical difference was observed in KYSE-140. Bioinformatics analysis revealed a positive correlation between MFAP2 expression and anti-tumor (M1 type) macrophages in EAC tissues, whereas in ESCC tissues, MFAP2 correlated positively with non-activated (M0 type) macrophages. RNA sequencing indicated that MFAP2 is involved in immune pathways and can promote PTGS2 expression. Collectively, this study preliminarily evaluates the function and potential molecular mechanism of MFAP2 in ESCC, offering new therapeutic targets and ideas for ESCC treatment.

## Introduction

Esophageal cancer (EC) is one of the most common malignant cancers worldwide, being the sixth leading cause of cancer-related death^1^, ^2^. EC primarily comprises two pathological types: esophageal squamous cell carcinoma (ESCC) and esophageal adenocarcinoma (EAC). Over 85% of EC cases worldwide are ESCC^3^. There are notable geographical differences in the distribution and incidence trends of these two types, with ESCC predominantly occurring in Southeast Asia and Central Asia, while EAC is more prevalent in North America, Australia, and Europe^4^. China is one of the countries with a high incidence of EC, and more than 90% of EC patients are diagnosed as ESCC^5^. Due to the lack of effective treatments, the five-year survival rate for ESCC patients remains below 30%^1^, underscoring the importance of exploring the molecular mechanisms underlying this disease.

Extracellular matrix (ECM), the non-cellular component within all tissues and organs, plays crucial roles in cell communication, adhesion and proliferation^6^, ^7^. Dysregulation of ECM organization is implicated in various diseases, including chronic obstructive pulmonary disease, spinal cord injury, Alzheimer's disease, obesity, and cancers^8^. During tumorigenesis, the ECM complex is established by various proteins such as collagen, elastin, fibronectin, laminin, hyaluronic acid, proteoglycans, glycoproteins^9^. As a crucial component of the tumor microenvironment, ECM deposition, remodeling, and cross-linking enhance tumor cell growth, survival, migration, epithelial-mesenchymal transition (EMT), angiogenesis, and immune evasion^10^. Emerging evidence suggests that the stiffened ECM in tumors significantly impairs the efficacy of chemotherapy, radiotherapy, targeted therapy, and immunotherapy^11^, making ECM components or related signaling pathway promising therapeutic targets for cancer.

Microfibrillar Associated Protein 2 (MFAP2), a glycoprotein of extracellular matrix, plays a vital role in the structure and organization of the ECM, particularly in microfibril formation^12^. As a member of the MFAPs family, MFAP2 is involved in numerous cellular processes, including cell adhesion, collagen fibrillogenesis, wound healing, vascular development, and metabolic diseases 13, 14. Recent studies have demonstrated that elevated MFAP2 levels play a significant role in the progression of several malignancies and serve as potential biomarkers or novel therapeutic targets for cancer^15^-^17^. In gastric cancer, increassed MFAP2 promotes tumorigenicity by modulating the integrin/FAK/ERK and TGFβ/smads signaling pathway^12^, ^18^. In colon cancer, silencing MFAP2 inhibits migration, invasion, and metastasis by inducing the degradation of DC-Like Kinase 3 (CLK3)^19^. Cui *et al.* found that MFAP2 is involved in variable shear factor QKI- circRNAs pathway through data mining analysis^20^. Although elevated MFAP2 expression has been observed in several cancers, including papillary thyroid cancer, hepatocellular carcinoma, ovarian cancer, melanoma, laryngeal squamous cell carcinoma, and breast cancer^21^-^26^, the expression and potential molecular mechanism of MFAP2 in different subtypes of esophageal cancer, particularly in ESCC, remain unclear.

## Materials and Methods

### Cell culture and transfection

10 human esophageal squamous cell carcinoma cell lines including KYSE-30, KYSE-70, KYSE-140, KYSE-150, KYSE-180, KYSE-270, KYSE-410, KYSE-450, KYSE-510 and KYSE-520 and an immortalized esophageal epithelial cell HET-1A were maintained and provided by Sino-British Research Centre for Molecular Oncology of Zhengzhou University. All cells were cultured in RPMI-1640 medium (Gibco) supplemented with 10% Fetal Bovine Serum (FBS) (PAN Seratech), 100 U/ml penicillin and 100 U/ml streptomycin at 37 °C in a humidified incubator with 5% CO2.

### Western blot

Cells were harvested and lysed with RIPA lysis buffer supplemented with protease inhibitors (Roche). 30 μg protein was separated using 8% SDS-PAGE and then transferred to a PVDF membrane (Amersham). After blocking with 5% non-fat milk for 1h, the membranes were incubated with primary antibodies overnight at 4°C. The primary antibodies were as following, anti-MFAP2 monoclonal antibody (sc-166075, Santa Cruz), anti-Flag tag monoclonal antibody (M20008, Abmart), anti-GAPDH antibody (60004-1-Ig, Proteintech). The membranes were then washed with TBST for three times and incubated with secondary antibody (ZB-2305, ZSGB-BIO) for 1.5 h. After washing, the membranes were visualized using enhanced chemiluminescence (ECL) (Thermo Scientific Pierce).

### Immunofluorescence

Control or MFAP2 overexpressing cells were plated on glass coverslips in 12-well plates. After 24h cells were fixed in 4% paraformaldehyde and blocked with goat serum for 1 hour at room-temperature. The cells were then incubated in Flag tag primary antibody overnight at 4°C. After washing, the cells were stained with secondary antibody conjugated with Alexa Fluor™ 4884 and 4', 6-diamidino-2-phenylindole (DAPI) (Thermo Fisher Scientific). Immunofluorescence images were acquired by a microscopy at an original magnification 200×.

### Immunohistochemistry

52 pairs of ESCC tissues sections were obtained from the Affiliated Hospital of Zhengzhou University, and complete clinical data were available for 50 patients (Table 1). All specimen were from surgical resection patients who had not undergone radiotherapy or chemotherapy. Sections were deparaffinized, rehydrated and blocked with goat serum. MFAP2 primary antibody was added to the sections and incubated overnight at 4°C. Slides were stained with EliVision plus kit and a 3'3-diaminobenzidine kit (MaiXin.Bio) according to the manufacturer's instructions. All patients provided informed consent, and the study was approved by the Ethics Committee of Affiliated Hospital of Zhengzhou University and the Ethics Committee of Zhengzhou University.

### MTS assay

Cells were seeded in 96-well plates at a density of 2000 cells per well. After 1 to 5 days, 10 μL MTS/PMS solution reagent (Progema) was added and incubated for 2 hours, then the supernatant was transferred to a new plate and the absorbance was measured with a spectrophotometer at OD490 nm. Cell proliferation curve was mapped using GraphPad software. All experiments were performed in triplicate and repeated three times.

### Colony formation

Cells (5000 cells/ well) were seeded in a 6-well plates and cultured for 7 days. The cells were then washed with PBS and fixed with 4% paraformaldehyde for 15 min, and stained with 0.1% crystal violet for 10 min. After additional washes with PBS, colonies were photographed using a camera, and the absorbance of eluted crystal violet was measured at 590 nm by adding 250 μL of 30% acetic acid.

### Wound healing assay

2×10^5^ cells were seeded into 12-well plates and cultured overnight. A straight line was then scraped across the cell layer using a 200 μL plastic pipette tip. The remaining cells were washed with PBS buffer and incubated in medium containing 1% serum. Images were captured using a microscope after 24 hours, and the percentage of wound closure was analyzed using Image J software.

### Transwell assay

1×10^5^ cells suspended with 100 μL serum free medium were seeded into the upper chambers of the transwell filter (Corning Costar), while the lower chambers were supplemented with 10% FBS medium. After 24 h, cells are fixed with 4% paraformaldehyde, stained with 0.1% crystal violet and photographed with a microscope. Subsequently, crystal violet was eluted with 200 μL 30% acetic acid and the absorbance was measured with a spectrophotometer at OD590 nm. For invasion assay, the upper champers were pre-coated with 100 μL Matrigel (BD) and cells were fixed after incubation 36 h.

### Xenograft assay

4-week old female BABL/C nude mice were purchased from Vital River Laboratory Animal Technology Co.Ltd. 1×10^6^ control and MFAP2 overexpressing cells were subcutaneous injection into both sides of back. 4 weeks later, all mice were sacrificed and tumor tissues were resected. The study was approved by the Animal Welfare and Research Ethics Committee of Zhengzhou University.

### Bioinformatics analysis

RNA expression and survival data of TCGA-ESCA including 89 EAC, 96 ESCC and 11 normal samples were downloaded from UCSC Xena. RNA expression data for MFAP2 in GSE161533 was obtained from NCBI GEO datasets and RNA sequencing data from 120 pairs ESCC samples were from our previous study^27^ (HRA000111, China National Center for Bioinformation). Differential gene expression analysis and heatmap clustering were performed using R studio. Overlapping genes were mapped with Venny 2.0 and protein interactions were predicted using STRING software. Comprehensive analysis of tumor-infiltrating immune cells was accomplished using TIMER and CIBERSORT.

### Statistical analysis

All statistics analysis were performed using GraphPad and SPSS software. One-way ANOVA and t-test were used to determine p-values. For overall survival curves, the Kaplan-Meier method and the log-rank test were used. Statistical significance was set at p < 0.05.

## Results

### The expression of MFAP2 in ESCC tissues was higher than normal and EAC

**Figure 1:**
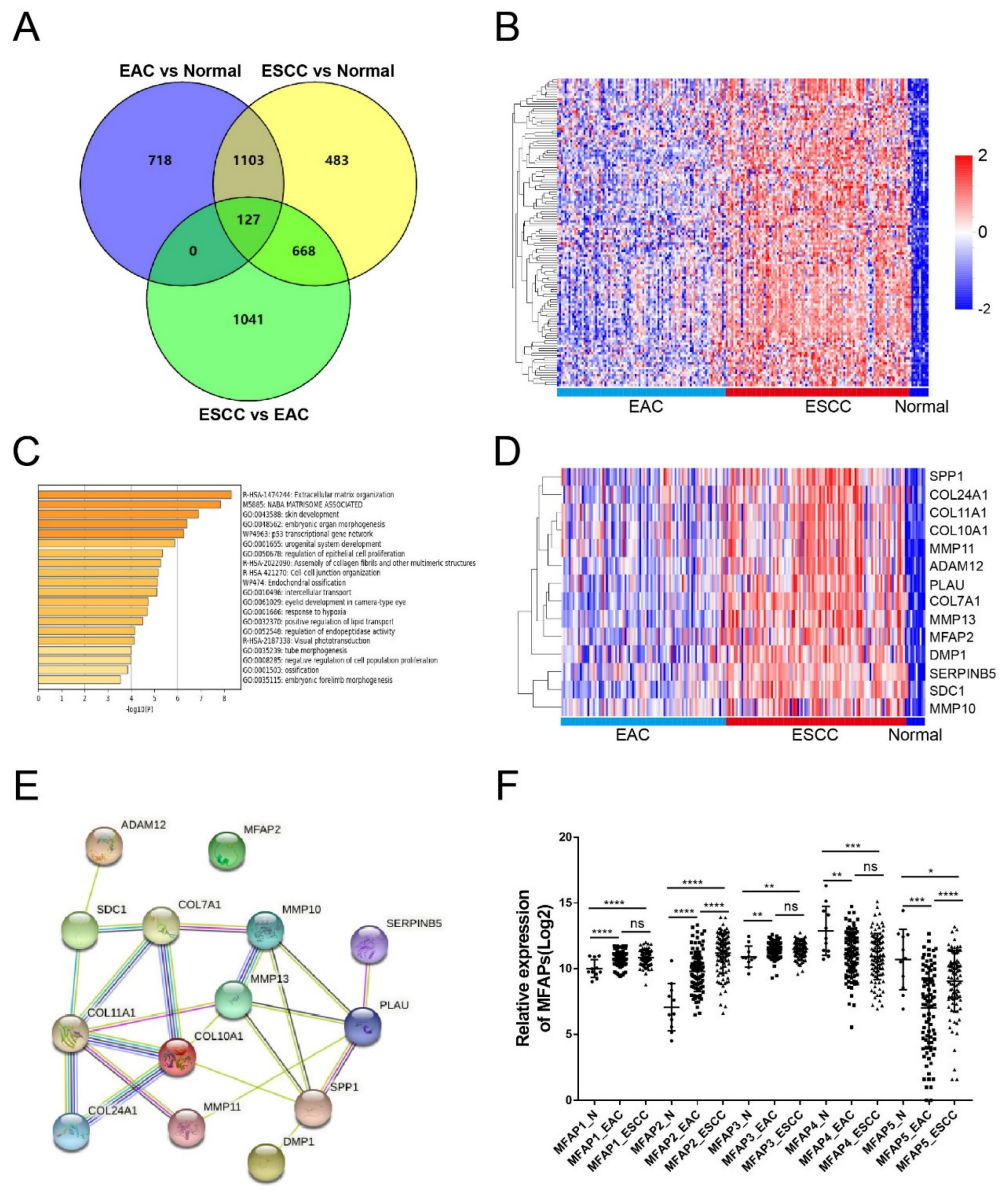
** The expression of MFAP2 in normal tissues and esophageal cancer.** A, Overlap analysis of increasing genes (fold change >2) in EAC and ESCC (TCGA database) with Venny software. B, The heatmap of 127 genes which were upregulated in ESCC samples comparing with normal and EAC tissues. C, The Gene Ontology (GO) enrichment of 127 genes by Metascape software. D, The heatmap of extracellular matrix organization pathway enriched genes in normal, EAC and ESCC tissues. E, The interaction network of extracellular matrix organization genes was analyzed with String software. F, Relative expression of MFAPs family including MFAP1, MFAP2, MFAP3, MFAP4 and MFAP5 in different tissues.

Considering that more than 90% of esophageal cancer patients were diagnosed as ESCC in China, to investigate the expression of MFAP2 in ESCC, we analyzed differentially expressed genes in EAC and ESCC tissues using TCGA-ESCA data. The overlapping genes (fold change >2) among EAC vs normal, ESCC vs normal and ESCC vs EAC were further analyzed with Venny software. As shown in Figure 1A, 718 genes were highly expressed only in EAC and 483 genes were significantly upregulated in ESCC. Impressively, 127 genes were upregulated in both EAC and ESCC compared to normal tissues, with higher expression in ESCC. This trend was also observed in the heatmap (Figure 1B). In order to clarify the major pathways of these 127 genes, enrichment analysis was performed using Metascape software. The results demonstrated these genes were enriched in pathways such as extracellular matrix organization, NABA matrisome associated, skin development, embryonic organ morphogenesis and regulation of epithelia cell proliferation (Figure 1C). Cluster and interaction analysis showed that genes involved in extracellular matrix organization pathway were highly expressed in ESCC (Figure 1D) and could form interacting complex other than MFAP2 (Figure 1E). Therefore, we further assessed the expression of MFAPs family in esophageal cancer tissues. As illustrated in Figure 1F, MFAP1, MFAP2 and MFAP3 were up-regulated in both EAC and ESCC, while MFAP4 and MFAP5 were down-regulated in cancer tissues, indicating that MFAP2 might play crucial role in ESCC.

### High MFAP2 was associated with the progression of ESCC patients

**Figure 2:**
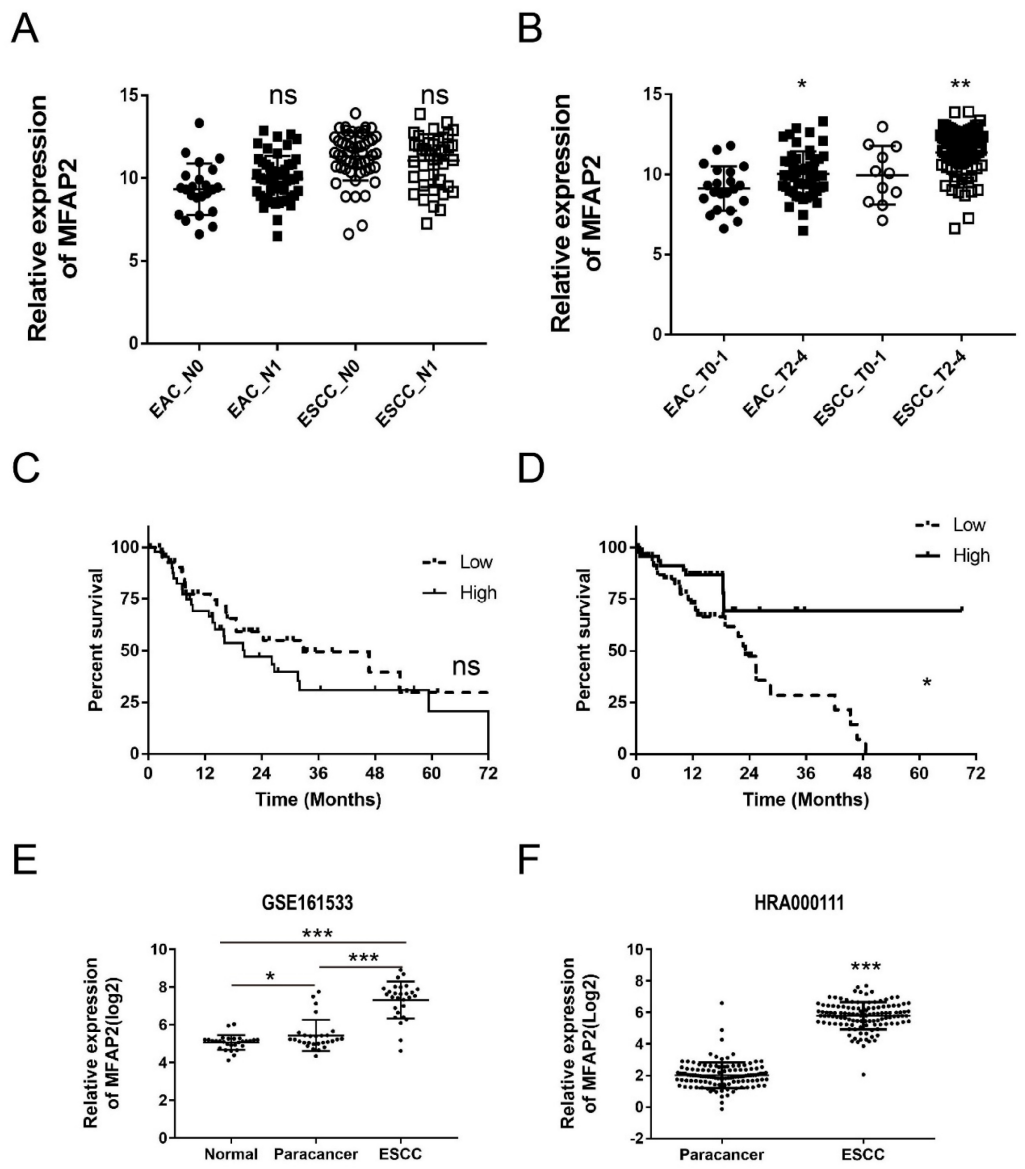
** The clinic significance of MFAP2 expression in EAC and ESCC.** A, Relative expression of MFAP2 in esophageal cancer with or without lymph node metastasis. B, Relative expression of MFAP2 in esophageal cancer with low (T0-T1) and high T stage (T2-T4) according to TNM stage. C, The overall survival of EAC patients with low or high MFAP2 expression. D, The overall survival of ESCC patients with low or high MFAP2 expression. E, The expression of MFAP2 in normal, paracancer and ESCC tissues was analyzed with GSE161533 datasets (GEO database). F, Relative expression of MFAP2 in 120 pairs of ESCC from this study (data were uploaded in China NGDC database).

To further evaluate the clinical significance of MFAP2 in ESCC, we systematically analyzed the relationship between MFAP2 expression and lymph node metastasis, tumor size and survival time. The results demonstrated that MFAP2 expression was not significantly different between metastasis and non-metastasis groups (Figure 2A), but was correlated with tumor stage (Figure 2B). Interestingly, MFAP2 expression in EAC was not associated with overall survival, whereas ESCC patients with high MFAP2 expression had worse prognoses (Figure 2C&D). Subsequently, we validated MFAP2 expression using a reported data (GSE161533) and our previous RNA sequencing data (HRA000111), and the data confirmed that MFAP2 was significantly upregulated in ESCC tissues.

### MFAP2 was mainly expressed in ESCC cancer cells

**Figure 3:**
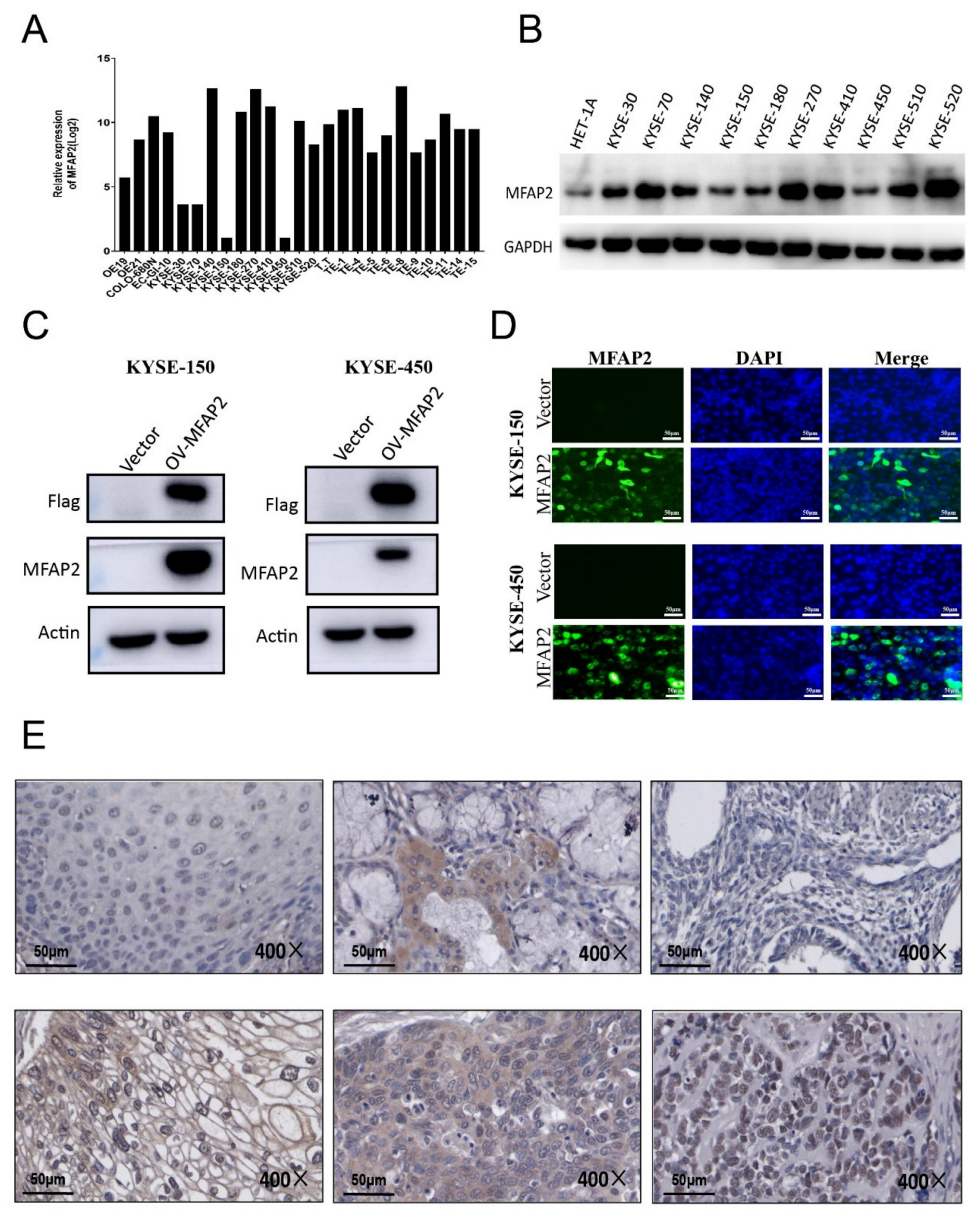
** The protein expression of MFAP2 in ESCC cell lines and tissues.** A, Relative RNA expression of MFAP2 in 25 ESCC cancer cell lines were analyzed using CCLE data (Xena database). B, Western blot assay was performed to evaluate the protein level of MFAP2 in 10 ESCC cancer cell lines and immortalized esophageal cell line HET-1A. C, MFAP2 overexpressing KYSE150 and KYSE-450 cell lines were constructed with lentivirus and protein level were validated by western blot. D, Protein expression and location of MFAP2 in overexpressing cell lines were analyzed with immunofluorescence assay. E, The protein expression in ESCC tissues was detected with immunohistochemistry assay.

Since solid tumor tissue was composed of tumor cells and various non-malignant cells such as fibroblasts, immune cells, adipocytes, endothelial cells^28^, we further detected MFAP2 expression in ESCC cell lines and tissues. Data from the Cancer Cell Line Encyclopedia (CCLE, Xena database) revealed MFAP2 expression was relatively low in the EAC cell line OE19 and some ESCC lines, including KYSE-30, KYSE-70, KYSE-150, and KYSE-450, but high in other ESCC lines (Figure 3A). Western blot showed the protein levels in KYSE-30, KYSE070, KYSE-140, KYSE-270, KYSE-410, KYSE-510 and KYSE-520 were higher than KYSE-150, KYSE-180, KYSE-450 and the immortalized esophageal cell line HET-1A (Figure 3B). We then constructed MFAP2-overexpressing KYSE-150 and KYSE-450 cell lines, and immunofluorescence assays confirmed that the protein was mainly distributed in the cytoplasm and partially in the nucleus (Figure 3C&D). Immunohistochemistry results showed that MFAP2 was located in extracellular matrix, cytoplasm, and nucleus, indicating the tumor microenvironment could alter MFAP2 localization in esophageal squamous carcinoma cells and may play non-extracellular matrix roles (Figure 3E).

### MFAP2 inhibited cell migration and invasion *in vitro*

**Figure 4:**
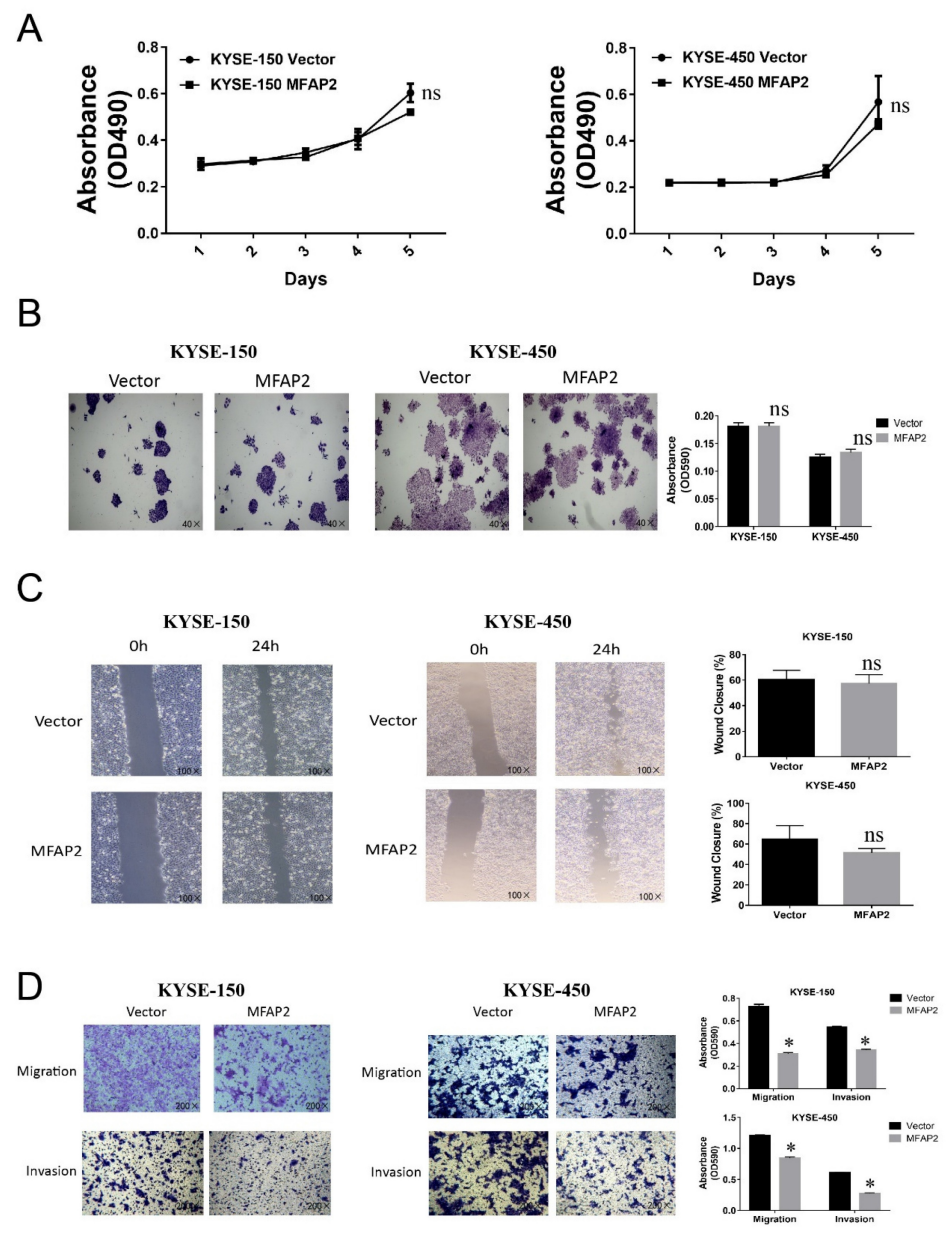
** The function of MFAP2 in ESCC cell lines.** A, The proliferation curve of control or MFAP2 overexpression cells was assessed by MTS assay. B, Clone formation assay was performed to validate the effect of MFAP2 on cell growth. C, The effect of MFAP2 on cell migration was analyzed by wound healing assay. D, Transwell assays with or without matrigel were conducted to evaluate the influence of MFAP2 overexpression on cell migration and invasion.

Several studies in gastric cancer, thyroid cancer, ovarian, colorectal cancer and hepatocellular carcinoma have demonstrated MFAP2 promoted cell proliferation and migration^18^, ^19^, ^21^, ^22^, ^29^, therefore we further investigated the function of MFAP2 in ESCC cell lines. Surprisingly, cell proliferation, clone formation and wound healing assays revealed that overexpressing MFAP2 had no significant effect on cell growth and healing activity (Figure 4A-B), whereas the Transwell assay demonstrated MFAP2 suppressed the migration and invasion abilities of ESCC cells (Figure 4D).

### MFAP2 promoted the growth of KYSE-450 cells in vivo

**Figure 5:**
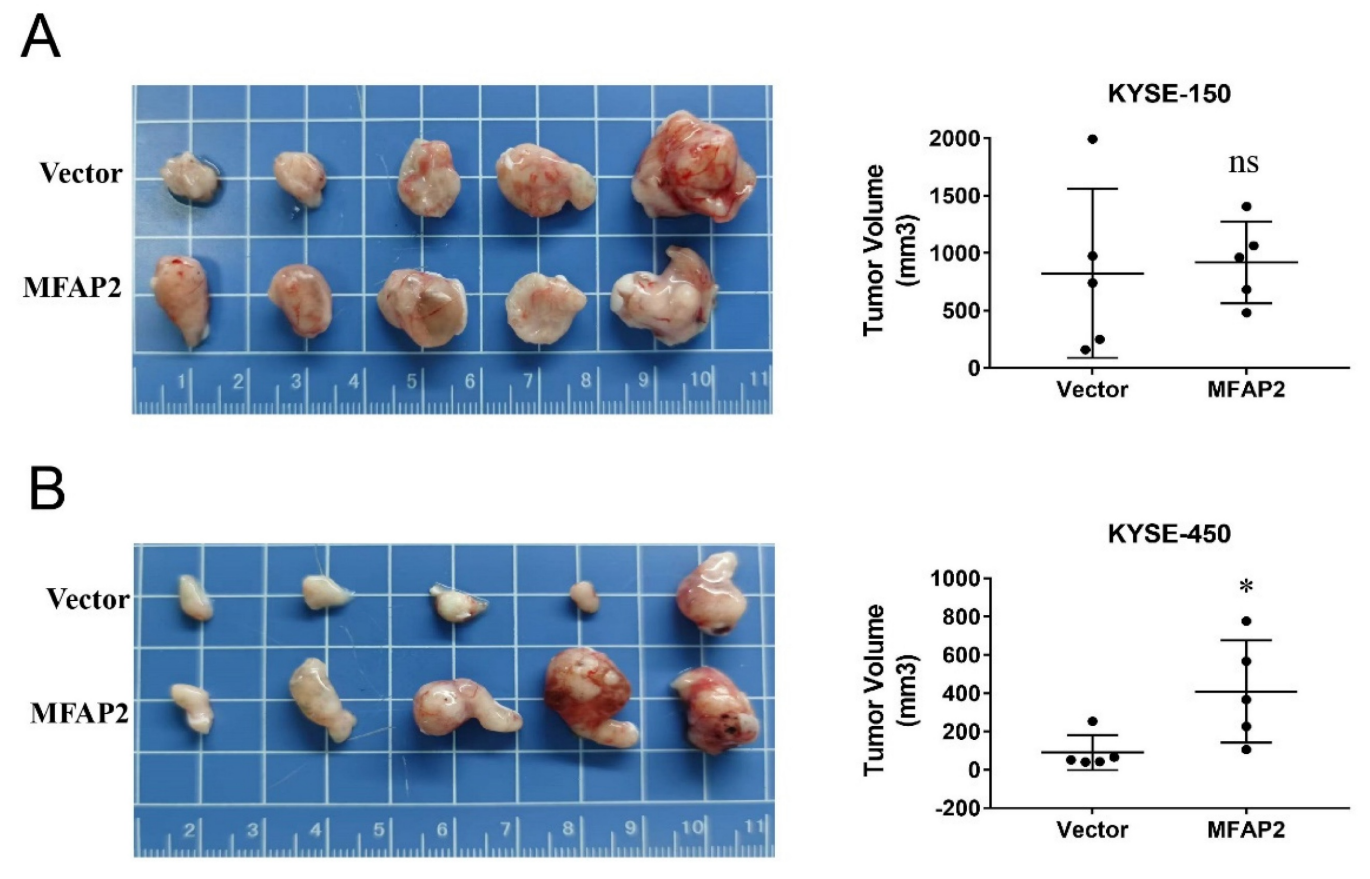
** The effect of MFAP2 overexpression on cell growth *in vivo*.** 1×10^6^ control and MFAP2 overexpressing cells were subcutaneous injection into BABL/C nude mice and the tumor volume of compared in KYSE-150 (A) and KYSE-450 (B).

Considering our previous clinical data showed that MFAP2 expression was related to tumor stage, we performed nude mice xenograft assays to better evaluate its role in ESCC. As shown in Figure 5, the tumors formed in overexpressing KYSE-450 cells significantly larger than control groups, whereas no remarkable difference was observed between overexpressing KYSE-150 and control cells. These results suggest that the function of MFAP2 may be associated with the tumor microenvironment and the genetic context of tumor cells.

### MFAP2 was involved in PTGS2 signaling pathway

**Figure 6:**
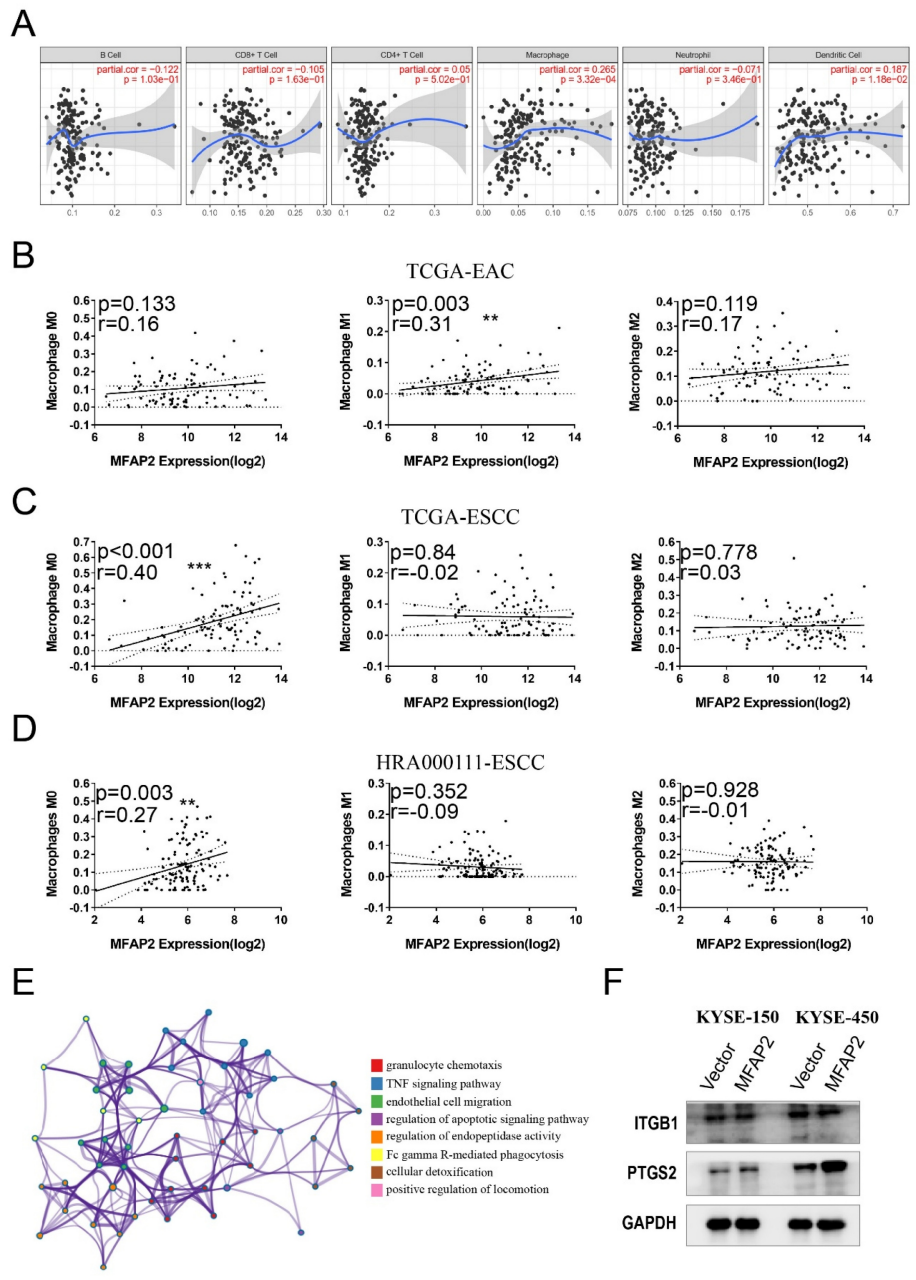
** MFAP2 was involved in PTGS2 signaling pathway.** A, The relationship between MFAP2 expression and immune infiltrates cells in ESCC tissues was analyzed with TIMER software. B, CIBERSORT tools was used to evaluate the correlation between MFAP2 and macrophage subtypes in EAC. C, The correlation between MFAP2 and macrophage subtypes in TCGA-ESCC. D, CIBERSORT analysis to evaluate the correlation between MFAP2 and macrophage subtypes in 120 ESCC tissues (this study). E, The enrichment analysis of upregulated genes in MFAP2 overexpression KYSE-450 cell lines using RNA sequencing data. F, Western blot assay was performed to detect the expression of potential downstream gene of MFAP2 including ITGB1 and PTGS2.

The inconsistency between *in vitro* and *in vivo* results suggests that the function of MFAP2 in ESCC might be related to the tumor microenvironment. To validate this hypothesis, we systematically analyzed immune infiltrates using TIMER software and found that high MFAP2 expression was positively correlated with macrophage infiltration but not with B cells, T cells, neutrophils, or dendritic cells (Figure 6A). Subsequently, CIBERSORT tools was used to evaluate the association between MFAP2 and different macrophage subtypes in EAC and ESCC. Impressively, no relevance was observed in EAC (Figure 6B), whereas MFAP2 was correlated with the M0 subtype in both TCGA-ESCC and our ESCC datasets (Figure 6C &D). RNA sequencing of KYSE-450 overexpressing cells demonstrated the elevated genes were enriched in pathways related to granulocyte chemotaxis, TNF signaling pathway, endothelial cell migration (Figure 6E). Western blot assays showed that overexpressing MFAP2 promoted PTGS2 expression in ESCC cell lines, particularly in KYSE-450 (Figure 6F). All these data indicated MFAP2 may aggravate tumor progression through PTGS2 signaling which has been proven to be a tumor driver.

## Discussion

Esophageal cancer primarily comprises two major subtypes: EAC, which arises from glandular cells in the lower esophagus and is associated with Barrett's esophagus, and ESCC, which usually occurs in squamous epithelial cells and is linked to high consumption of hot foods and alcohol^3^. During the past decades, advancements in high-throughput technologies have greatly enhanced our understanding of the molecular bases of EAC and ESCC. For EAC, whole-genome sequencing has identified several driver genes, including TP53, CDKN2A, SMAD4, ARID1A, ERBB2, KRAS, PIK3CA, SMARCA4, CTNNB1, ARID2, PBRM1, and FBXW7, which are frequently mutated^30^, ^31^. Additionally, invasive EAC tissues exhibit increased copy numbers of genes such as GATA4, KLF5, MYB, PRKCI, CCND1, FGF3, FGF4, FGF19, and VEGFA and loss of genes including FHIT, WWOX, PDE4D, PTPRD, and PARK2^32^. In ESCC, the main mutated genes of ESCC were most involved in cell cycle and apoptosis regulation, including TP53 (93%), CCND1 (33%), CDKN2A (20%), NFE2L2 (10%) and RB1 (9%).

Moreover, genes associated with histone modifier genes such as KMT2D (19%), KMT2C (6%) KDM6A (7%), EP300 (10%), CREBBP (6%) and those in the Notch pathway, including FAT1-3 (27%), NOTCH1-3 (22%), AJUBA (7%), FBXW7 (5%)^33^, ^34^, are also frequently mutated. In this study, we analyzed the differentially expressed genes in EAC and ESCC tissues using TCGA-ESCA data and identified 718 and 483 genes that are highly enriched in EAC and ESCC, respectively. We also found 127 of the 1230 shared genes that were significantly upregulated in ESCC compared to normal esophageal samples and EAC. These genes were primarily clustered in pathways related to extracellular matrix organization, NABA matrisome associated, skin development, embryonic organ morphogenesis, and regulation of epithelial cell proliferation. This finding is consistent with recent comparative research^35^.

MFAPs are important extracellular matrix glycoproteins involved in microfiber assembly, elastin production, and tissue environmental stability^17^. A total of five family members are identified, with MFAP2, MFAP4, and MFAP5 mainly expressed in osteoblastic-like cells, and MFAP1 and MFAP3 ubiquitously expressed^14^. Studies in deficient mice have revealed that MFAP2 plays a crucial role in skeletal development, hemostasis, and lipid uptake by regulating TGF-β signaling^36^. In tumors, increased MFAP2 expression has been validated in papillary thyroid cancer, hepatocellular carcinoma, ovarian cancer, melanoma and laryngeal squamous cell carcinoma^21^-^26^. Recently, Cui Y *et al.* speculated that the variable shear factor QKI is involved in EMT process by influencing certain circRNAs that may target MFAP2, and Qiu *et al.* revealed that MFAP2 expression was elevated in several cancer tissues through data mining^20^, ^37^. This study demonstrated that MFAP2 expression in ESCC was significantly higher than in normal esophageal samples and EAC. Although the homologous subfamily members MFAP1 and MFAP3 were elevated in both ESCC and EAC, no difference was observed between esophageal cancer subtypes. In contrast to MFAP2, the expression of MFAP4 and MFAP5 was downregulated in cancer tissues.

It has been reported that MFAP2 promotes the progression of malignant tumors through multiple processes. In melanoma, knockdown of MFAP2 inhibits B16 melanoma cell migration, invasion, and the protein expression of EMT biomarkers^24^. Interfering with MFAP2 in gastric cancer disrupts the integrin/FAK/ERK and TGFβ/Smads signaling pathways^12^, ^18^. In colon cancer, silencing MFAP2 induces the degradation of CLK3 and restrains the migration of HCT-116 and RKO cell lines^19^. This study showed that although MFAP2 expression was elevated in ESCC and high levels of MFAP2 were closely related to poor patient prognosis, overexpressing MFAP2 in ESCC cells only enhanced tumor growth *in vivo* rather than *in vitro*. Further experiments demonstrated that MFAP2 expression was correlated with the infiltration of the M0 subtype in ESCC, and this correlation may be associated with the PTGS2 signaling pathway.

## Figures and Tables

**Table 1:** The clinicopathological characteristics of gastric cancer patients

Parameters	No.
Age (yrs)	
<65	33
≥65	17
Gender	
Male	32
Female	18
Size (maximal diameter)	
<5cm	16
≥5cm	34
Depth of invasion (pT)	
T_1_, T_2_	16
T_3_, T_4_	34
Lymph node status (pN)	
N0	28
N1-3	22
Pathological stage (pStage)	
Stage I-II	29
Stage III-IV	21

## Abbreviations

ESCC: esophageal squamous cell carcinoma
EAC: esophageal adenocarcinoma
MFAP2: microfibril associated protein
ECM: extracellular matrix
TCGA: the cancer genome atlas
GEO: Gene Expression Omnibus
MTS: 3-(4,5-dimethylthiazol-2-yl)-5-(3-carboxymethoxyphenyl)-2-(4-sulfophenyl)-2H-tetrazolium
